# Tissue dissolving ability of several endodontic irrigants on bovine pulp tissue

**Published:** 2007-07-05

**Authors:** Abbasali Khademi, Ehsan Usefian, Mahboobe Feizianfard

**Affiliations:** 1 Departmentof Endodontics, Dental School, Isfahan Medical University, Isfahan, Iran., Dental School, Isfahan Medical University, Isfahan, Iran.; 2 General Practitioner.

**Keywords:** Chlorhexidine gluconate, MTAD, Sodium hypochlorite, Tissue dissolving ability

## Abstract

Introduction: A desirable characteristic of root canal irrigants is the ability of dissolving soft tissues. Sodium hypochlorite, an antibacterial and tissue solvent irrigant used in endodontic treatment is known to be toxic for periapical tissues. Chlorhexidine gluconate, an effective antimicrobial agent, is another irrigant with limited tissue dissolving ability. A mixture of a tetracycline isomer, an acid, and a detergent (MTAD), has recently been introduced as an alternative irrigant in root canal therapy. The purpose of this study was to evaluate the tissue dissolving effect of these root canal irrigants on bovine pulp tissue.

Materials and Methods: Fifty pieces of bovine pulp tissue 80 mg each were treated with either normal saline, MTAD, 2% chlorhexidine gluconate, 2.6% NaOCl or 5.25% NaOCl for 10 min at 37°C. Desiccated pre-treatment and post-treatment weights of samples were compared. Using Kruskal-Wallis and Mann-Whitney U tests, data was analyzed.

Results: Tissue dissolution effect of 5.25% NaOCl (85.98%) was statistically greater than that of all other solutions (P<0.05). Chlorhexidine gluconate had the weakest dissolution effect, and dissolved only 9.36% of the sample tissue. No significant differences were observed between the dissolution effects of normal saline or MTAD.

Conclusion: Based on the results of this study, the use of NaOCl as intracanal irrigation during instrumentation is recommended, because of its greater tissue dissolution effect.

## Introduction

Root canal preparation is accomplished using a combination of mechanical instrumentation and chemical irrigation (1). Due to anatomical complexities, mechanical action of instruments alone is unable to reach all areas of the root canal system. Irrigating solutions are therefore important in extending debridement into the physically inaccessible areas (2). Being poten-tially able to enhance root canal cleansing, tissue dissolving capability of any irrigating solution is important (3). Offering good tissue dissolving capacity and being antibacterial, sodium hypochlorite (NaOCl) is historically the most favored irrigant in endodontics (4). However, NaOCl is cytotoxic at recommended concentrations of 2.6% or 5.25 % (5). 

Chlorhexidine gluconate is a widely used antibacterial agent in dentistry. It possesses a broad-spectrum antimicrobial action (6), substantivity (7) and a relative absence of toxicity (6). These properties have led to the suggestion using this solution as an irrigant in endodontics. 

MTAD, (a mixture of a tetracycline isomer [Doxycycline], an acid [Citric acid], and a detergent [Tween 80]), is now marketed as a final rinse to remove smear layer from the surface of instrumented root canals (8). MTAD is reported to be less cytotoxic than 5.25% NaOCl, Ca(OH)_2_ paste, Peridex and ethylene-diaminetetraacetic (EDTA) acid, but more cytotoxic than 2.63%, 1.31% and 0.66% NaOCl (9). 

MTAD has a higher antibacterial efficacy than 5.25% NaOCl in prepared roots contaminated by whole salvia (10). MTAD is significantly more effective in killing *Enterococcus faecalis* than NaOCl when the latter is diluted to 1% or 0.5% (11). 

Beltz *et al.* (12) investigated the solubilizing action of MTAD, NaOCl and EDTA on bovine pulp and dentine. Their results indicated that tissue dissolving ability of MTAD was similar to that of 17% EDTA, but less than 2.6%, 5.25%, and 1.3% NaOCl.

The present study aimed to evaluate and compare the tissue dissolving properties of 2.6% and 5.25% NaOCl, 2% chlorhexidine gluconate, MTAD and normal saline on bovine pulp tissue**.**

## Materials and Methods

The posterior teeth of three fresh bovine mandibles were split into halves by a high speed rotary device, their fresh pulps removed and rinsed thoroughly under tap water to remove blood and debris followed by storage and freezing at -5°C. The frozen pulp tissues were first broken into smaller pieces with a mallet prior to testing the dissolving capacity of the five different irrigants used in this study. 

Test solutions used were 2.6% and 5.25% NaOCl (Merck, Germany), 2% chlorhexidine gluconate (Medichem, Spain), normal saline (Shahid Ghazi Pharmaceutical, Iran), and MTAD freshly prepared before each session, according to the proprietary protocol of Loma Linda University, (doxycycline HCl, citric acid, and Tween 80, all purchased from Sigma-Aldrich Co., St. Louis, MO).

Squares of Wathman ashless filter paper cut to 1.5×1.5 cm were used to hold each of the 50 tissue samples which were subsequently allocated to one of the 5 groups of 10 specimens for each irrigant. The 50 paper squares were first desiccated in a glass container with calcium chloride crystals for 24h; then numbered and randomly allocated one of the five test groups. Each single paper square was placed on an analytical balance with ±0.001 g accuracy (Scaltec-Germany) and the readout recalibrated to zero. Pulp tissue was added until each paper contained 80 mg (±1mg) of bovine pulp tissue. Each group of 10 paper/tissue samples for testing one irrigant was placed into a desiccator at 37°C for 24h prior to re-weighing each to determine their individual desiccated pre-treatment weight. Three weight readings were made by a same investigator for each specimen and the average weight recorded to the nearest 0.1 mg. Each paper/tissue sample was then placed in an individual beaker and rehydrated by immersing in 1 liter of distilled water for 30 minutes immediately prior to a dissolution trial. 

Each sample was removed from the beaker, drained of excess water on a dry paper towel and then placed into empty first well of 5-cell well plate (Isfahan Dental School) similar to that used in the study of Morgan *et al.* (13) and placed on the platform of a Shaker Incubator F.F 81 (Pars Azma, Iran) at 37°C and 5% humidity. Ten mLs of test solution was added to same well and agitated for exactly 90s at the maximum speed (200 rpm), in an effort to produce fluid movements during irrigation of a root canal. Then filter/tissue sample was removed from the device when agitation stopped and it was placed on a dry paper towel for 30s to allow bulk excess test solution to drain off. The filter/tissue sample was placed into second well and 90s/30s cycle was repeated by fresh solution. This procedure was repeated for 5 times in other wells of 5-cell well plate. The total time for specimen/test solution contact was therefore 7.5 mins (5×90 s) and the total time for testing each sample was 10 mins (5×120 s). 

The sample was then immediately placed back into a beaker of distilled water for 30 min in order to arrest any residual solution activity affecting the tissue. Testing the 10 samples of each test group, they were returned to a desiccator at 37°C for 24 hrs before their desiccated post treatment weights were re-measured on a scale, as per their pretreatment weight determination. The mean percentage weight loss was calculated for each sample using the following equation: Weight loss % = [Initial weight (mg) – Final weight (mg)]/ Initial weight (mg) x 100.

The trials for all specimens of each group were done at the same day. Using Kruskal-Wallis and Mann-Whitney U tests, data was analyzed.

## Results

**Table 1:** Mean weight change of bovine pulp after treatment

**Test solution**	**Mean Weight loss (%)**	**SD**
**5.25% NaOCl**	**85. 98**	**17. 74**
**2.6% NaOCl**	**68.60**	**12.95**
**Normal saline**	**36. 66**	**17. 06**
**MTAD**	**34. 46**	**24. 62**
CHX*	**9.36**	**11. 69**

*2% Chlorhexidine gluconate

The mean tissue weight loss was calculated for all 10 samples of each test group. The Kruskal-Wallis test was performed to compare groups since group variances were not normally distributed (Table-1). Group pairs were then compared using the Mann-Whitney U test for statistical significance (Table 2). A Kruskal-Wallis test was used for analysis, because the Bartlett test showed the group variances were not homogenous. The Kruskal-Wallis test also indicated a statistically significant difference between the mean percentage weight change values in five groups (P= 0.05). 

Pairs of groups were compared using the Mann-Whitney U test. This showed that all treatment pairs except normal saline and MTAD had statistically significant differences in their mean tissue weight loss amounts (Table-2).

## Discussion

Bovine pulp tissue was used in this study to simulate human pulp tissue similar to previous studies (12, 14-16). Koskinen *et al.* (17) showed that bovine pulp was histologically similar to the human pulp. Bovine tendon, collagen, rat dermal connective tissue and bovine muscle have been used to simulate the human pulp tissue in other studies on efficacy of root canal irrigants (3, 18, 19). But variables such as different experimental conditions (concentration, pH, volume, mechanical agita-tion, exchange/refreshment solution, physical irrigation, surface area of tissue exposed to test solution, exposure time, and temperature) can affect tissue dissolution capacity of a test solution (19, 20). 

**Table 2:** Group pairs comparison by Mann-Whitney U test (P-values

**Group**	**Normal saline**	**5.25% NaOCl**	**2.6% NaOCl**	CHX*
**MTAD**	**0.684**	**0.000**	**0.007**	**0.003**
**Normal saline**	**٭**	**0.000**	**0.000**	**0.000**
**5.25% NaOCl**	*	*	**0.019**	**0.000**
**2.6% NaOCl**	*	*	*	**0.000**

*2% Chlorhexidine gluconate

The results of the current study show that the tissue-dissolving capacity of 5.25% NaOCl (85%) was greater than that of 2.6% NaOCl (68%). This was in agreement with the results of Hand *et al.* (21) and Abou-Rass *et al.* (19), 

but conflicts the results reported by Beltz *et al.* (12) and Gordon *et al.* (15). 

Data of current study showed that chlorhex-idine gluconate (9.36%) has very little tissue dissolution capacity, which is in agreement with the findings of Baumgartner *et al.* (22) and Naenni *et al.* (23). Beltz *et al.* (12) investigated the percentage of tissue weight loss after exposing bovine pulp and dentin to various concentrations of NaOCl, 17% EDTA or to MTAD. Their results showed that 2.6% and 5.25% NaOCl dissolved 90% of a lyophilized pulp specimen, and that the dissolution effects of EDTA (51.5%) were similar to those of MTAD (49.3%) and isotonic saline (62%). 

It appears that, in the current study, NaOCl dissolves bovine pulp effectively but that chlorhexidine gluconate displays very little tissue-dissolution capacity. The dissolution effect of MTAD (34.5%) in our study is similar to that of normal saline (36.66%). Thus, if MTAD or chlorhexidine gluconate used as a final rinse, it would be also necessary to use NaOCl during instrumentation for the purpose of pulpal debris removal. Torabinejad* et al. *also recommend regime of 1% NaOCl for removing the organic portion of smear layer before MTAD use as a final rinse. As a result, the inorganic component of smear layer is removed without significant effect on dentinal tubule structure (24).

## Conclusion

Based on the results of our present investigation, the use of NaOCl for intracanal irrigation during instrumentation must show the highest tissue dissolving capacity. MTAD and normal saline can be expected to have a similar dissolution capacity which would be lower than NaOCl. Chlorhexidine gluconate showed the lowest dissolution capacity.
